# Mosaic analysis and tumor induction in zebrafish by microsatellite instability-mediated stochastic gene expression

**DOI:** 10.1242/dmm.014365

**Published:** 2014-01-30

**Authors:** Wouter Koole, Marcel Tijsterman

**Affiliations:** Department of Toxicogenetics, Leiden University Medical Center, Leiden, 2333 ZC, The Netherlands.

**Keywords:** Mosaic analysis, Microsatellite instability, Lineage tracing, Tumor induction

## Abstract

Mosaic analysis, in which two or more populations of cells with differing genotypes are studied in a single animal, is a powerful approach to study developmental mechanisms and gene function *in vivo*. Over recent years, several genetic methods have been developed to achieve mosaicism in zebrafish, but despite their advances, limitations remain and different approaches and further refinements are warranted. Here, we describe an alternative approach for creating somatic mosaicism in zebrafish that relies on the instability of microsatellite sequences during replication. We placed the coding sequences of various marker proteins downstream of a microsatellite and out-of-frame; *in vivo* frameshifting into the proper reading frame results in expression of the protein in random individual cells that are surrounded by wild-type cells. We optimized this approach for the binary Gal4-UAS expression system by generating a driver line and effector lines that stochastically express Gal4-VP16 or UAS:H2A-EGFP and self-maintaining UAS:H2A-EGFP-Kaloop, respectively. To demonstrate the utility of this system, we stochastically expressed a constitutively active form of the human oncogene H-RAS and show the occurrence of hyperpigmentation and sporadic tumors within 5 days. Our data demonstrate that inducing somatic mosaicism through microsatellite instability can be a valuable approach for mosaic analysis and tumor induction in *Danio rerio*.

## INTRODUCTION

Somatic mosaicism is a widely used term to describe the presence of two genetically different cell populations in a single individual. Mosaic animals arise from genetic alterations or epigenetic changes (e.g. X-chromosome inactivation) in a subset of cells during development. Mosaicism can also be obtained when cells are transplanted from one animal to another, although technically this is termed chimerism. The importance of mosaic analysis was evident from the moment the first naturally occurring mosaic animals were discovered, nearly 100 years ago (examples are given in Xu and Rubin, 2012). Since then, investigators have developed various techniques to stimulate somatic mosaicism, enabling experiments to trace cell lineage and the study of developmental processes and gene function in whole animals (for an overview of established techniques, see Buckingham and Meilhac, 2011).

An advantage of mosaic analysis in the zebrafish over other model organisms is that the development of the embryos occurs *ex utero* and the embryos are transparent. Therefore, individual cells can be studied during embryonic development and imaged *in vivo* with relative ease. Additionally, the identification of ‘casper’ fish (White et al., 2008), which are almost entirely transparent because of the lack of two pigment cell types – melanocytes and iridophores, allows live imaging in adult animals.

Several methods exist in zebrafish to create mosaic animals (for reviews, see Blackburn and Langenau, 2010; Carmany-Rampey and Moens, 2006; Weber and Köster, 2013). For example, transplantation assays and DNA and/or mRNA injection at the one-cell stage can be used, but these are invasive, time-consuming and often technically challenging; therefore, non-invasive genetic approaches are preferred. In the last few years, several such approaches have been developed (Boniface et al., 2009; Collins et al., 2010; Emelyanov and Parinov, 2008; Esengil et al., 2007; Gerety et al., 2013; Hans et al., 2011; Hans et al., 2009; Knopf et al., 2010; Pan et al., 2011), most of which rely on Cre recombinase-controlled lox site recombination (Cre-lox system), and are controlled either through heat shock or administration of the ligand 4-hydroxytamoxifen (4-OHT). Despite their promise and advances, these techniques also have limitations and drawbacks, such as the leakiness of the estrogen receptor variant (ER^T2^) that is used to modulate Cre (Boniface et al., 2009; Gerety et al., 2013; Mosimann and Zon, 2011) and the known toxicity and side-effects of Cre recombinase and the drug 4-OHT (Anastassiadis et al., 2010; Schmidt-Supprian and Rajewsky, 2007). Furthermore, there are reservations as to whether these drugs can penetrate all tissues, especially in adult fish. Moreover, the available number of Cre-lox lines in zebrafish is currently limited and restricts the application of these systems.

The binary Gal4-UAS expression system is a powerful, and commonly used, transgenic tool in the zebrafish. Since the introduction of the Gal4-UAS system in zebrafish more than a decade ago (Scheer and Campos-Ortega, 1999), hundreds of so called ‘driver lines’ have become available that express the transcriptional activator Gal4 under the control of a specific enhancer or promoter. Furthermore, the repertoire of ‘effector-lines’, which express Upstream Activated Sequence (UAS)-linked transgenes in specific tissues when activated by Gal4 binding to the UAS, is large and rapidly expanding. Animals that make use of this system express a specific gene in all cells of a certain type of tissue (depending on the Gal4-driver and UAS-effector line), and the surrounding tissues remain wild type. However, the ability to trace a single (often mutant) cell within a wild-type tissue is preferred for cell lineage tracing, gene function experiments and cancer modeling studies. To achieve this goal, we developed a system in which single cells express a gene – e.g. *Gal4* or oncogenic *H-RAS* – only when the ORF is placed in-frame after an *in vivo* frameshift mutation. Here, we show that the random activation of genes through microsatellite instability can be a valuable tool for mosaic analysis in zebrafish.

**RESOURCE IMPACT**

**Background**

The zebrafish is an elegant and powerful vertebrate model system that is increasingly being used to study diseases and their underlying molecular mechanisms. Its small size, its fast rate of reproduction, the ease and relatively low costs of culturing, its striking anatomical and physiological similarities to mammals, and its transparency make the zebrafish a valuable model in which to study human diseases and to test drugs. However, mostly because of a lack of appropriate reagents and technology, the use of zebrafish as a model system in which to mark individual cells that are genetically different from surrounding cells and then follow their fate has been under-addressed. Such an experimental model would be particularly relevant to the study of cancer, which is a process in which single cells grow out to become malignant tumors through a process of stochastic mutations followed by selection for increased growth within an organism that is itself not genetically compromised or challenged.

**Results**

In this article, the authors describe a technology to genetically alter and then trace individual transformed cells in living zebrafish. They show that genes can be stochastically activated *in vivo* by placing their coding sequences out-of-frame downstream of microsatellite sequences, which are prone to frameshifts during DNA replication. Because the gene of interest is initially out-of-frame, low frequency *in vivo* stochastic frameshifting activates the gene of interest in occasional cells, which can be traced if the gene of interest is tagged with a fluorescent marker protein. Thus, using this approach, the fate of single altered cells that are surrounded by wild-type cells can be determined in living animals. The authors demonstrate that this method can also be used to mimic tumor development. Specifically, they show that microsatellite-dependent stochastic activation of oncogenic H-RAS results in the formation of tumors within 5 days.

**Implications and future directions**

This study describes a new model system in which to trace single cells in living animals and to induce and monitor tumorigenesis. Because of its modular nature, this system can be easily adapted to study any (disease-related) protein of interest. Thus, the technology can be used to study and monitor the oncogenic effect of any (onco)gene of choice. Moreover, the fish system will also facilitate the search for cognate drugs, thus ultimately leading to a better understanding of the pathology of cancer and other diseases, and to the development of new therapeutics.

## RESULTS

### Microsatellite-dependent mosaicism

To investigate whether we could activate genes stochastically *in vivo* through microsatellite instability, we designed various reporter constructs in which we placed the coding sequence of LacZ downstream of a microsatellite-containing ORF. The constructs were designed in such a way that a frameshift-mutation within the microsatellite could bring the coding sequence of LacZ in-frame with the upstream ORF, for which we used the coding sequence of the enhanced green fluorescent protein (EGFP).

**Fig. 1.**
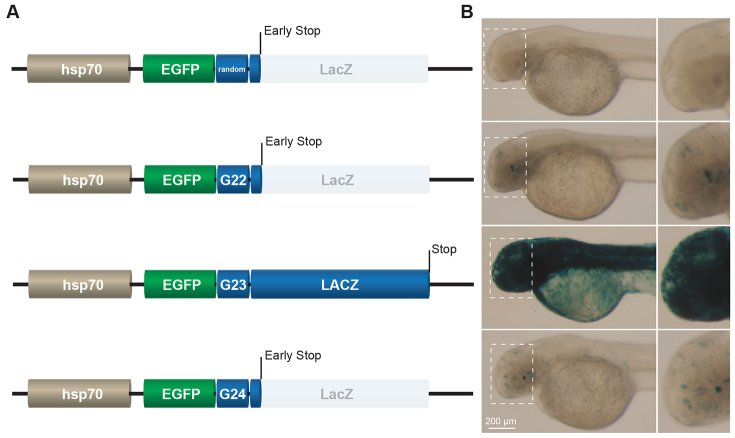
**Microsatellite-dependent stochastic gene activation in *Danio rerio***. (A) Schematic representation of reporters in which the coding sequence of LacZ was placed downstream of an EGFP ORF; a random sequence or a frameshift-prone G_22_, G_23_ or G_24_ microsatellite tract was placed in between these two elements. The random sequence, G_22_ and G_24_ put the LacZ ORF out-of-frame. The reporter was under the control of the *hsp70* promoter. (B) Images of LacZ-stained embryos (2 dpf) that had been injected with the corresponding reporters shown in A. The embryos show microsatellite-dependent stochastic LacZ expression (blue) *in vivo*. One-cell stage embryos were rendered transgenic by using Tol2-transposase-mediated transgenesis. Inset images on the right correspond to the dashed box areas shown in the left-hand images.

As a control, we placed the coding sequence of LacZ out-of-frame downstream of a random sequence (5′-GATTCTGCCAAGT-3′) that was not prone to frameshift mutations (Fig. 1A, upper reporter). Using Tol2-transposase-mediated transgenesis, which causes transient expression (Balciunas et al., 2006; Kawakami et al., 2004), we injected this reporter into one-cell stage embryos and analyzed the embryos for staining of LacZ two days later. We did not find any LacZ staining in any of the embryos that had been injected (*n*=100) (Fig. 1B, upper image). Although the upstream coding sequence of EGFP was in-frame, no EGFP expression was detected either, probably because the out-of-frame sequence of LacZ did not serve as a proper 3′UTR (comparable observations have been made in human cells; Koole et al., 2013). Next, we injected similar reporters in which we had replaced the random sequence with microsatellites that comprised 22, 23 or 24 guanines (G_22_, G_23_ and G_24_, respectively) resulting in reporters that had the ORF of LacZ in all three reading frames (Fig. 1). The length of these microsatellites were chosen based on experiments in human cells, which have shown that microsatellites with a similar tract-length are highly prone to frameshift mutations in wild-type cells (Koole et al., 2013). Embryos that had been injected with an in-frame LacZ coding sequence displayed LacZ-staining in the majority of cells (Fig. 1, microsatellite G23). Importantly, in contrast with the reporter that contained a random sequence, we observed stochastic expression of LacZ when its ORF was placed out-of-frame downstream of a microsatellite that comprised 22 or 24 guanines (Fig. 1). Different animals had different LacZ spots, both in terms of location and in the number of cells per spot, which reflects the stochastic nature of microsatellite-dependent gene activation. To further substantiate that reporter activation is the result of microsatellite instability, we assayed LacZ restoration in the reporters when they were injected into mismatch-repair-deficient animals (Feitsma et al., 2007). Previously, we have shown that the rate of frameshifts at microsatellites increases profoundly in mismatch-repair-defective *Caenorhabditis elegans* and human cells, using a similar type of microsatellite instability reporters (Koole et al., 2013; Pothof et al., 2003). Indeed, we also found increased rates of reporter activation in mismatch-repair-compromised zebrafish embryos (supplementary material Fig. S1), which provides strong support for causative frameshift events. Taken together, these data indicate that placing the coding sequence of a gene out-of-frame downstream of a microsatellite results in its stochastic expression *in vivo* due to microsatellite instability.

### UAS-effector lines that stochastically express genes

**Fig. 2.**
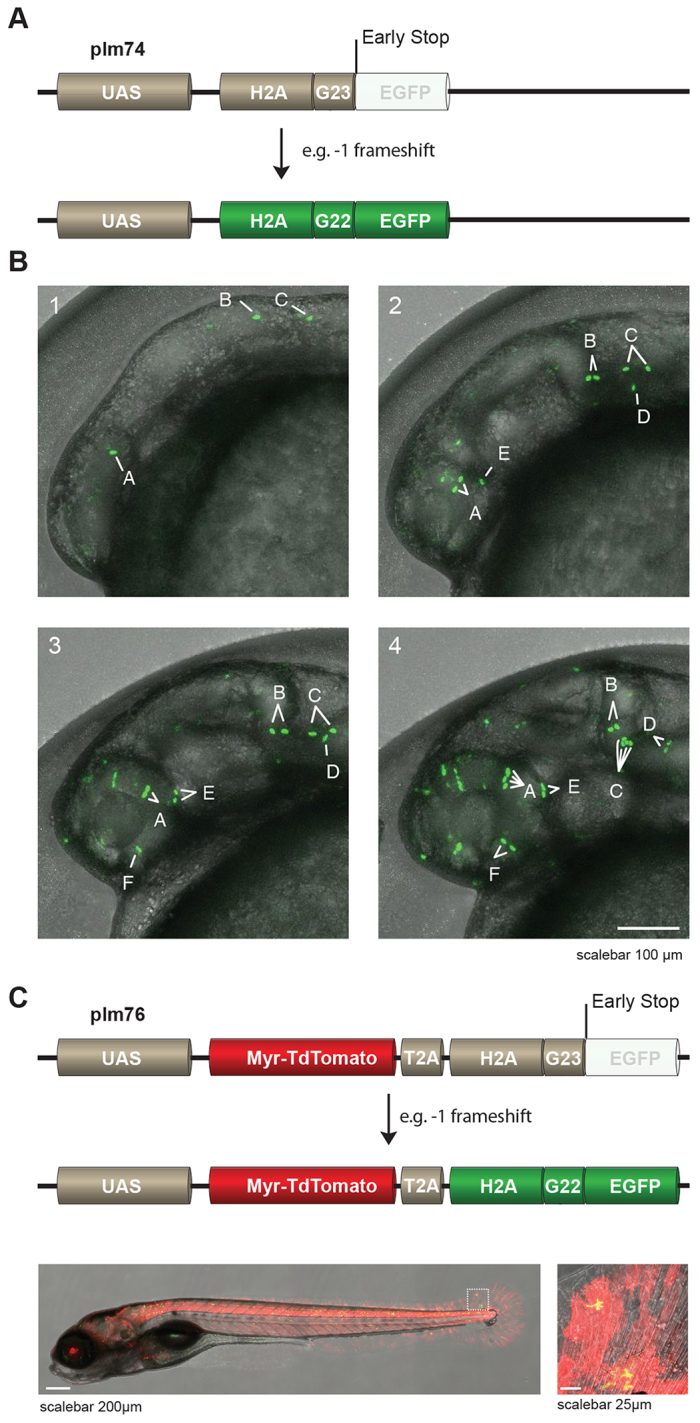
**UAS-reporters stochastically express H2A-EGFP**. (A) (Upper panel) Schematic representation of reporter plm74 in which frameshifts at a G_23_ microsatellite can result in EGFP expression. Note that, for illustration purposes, we used the example of a −1 frameshift, however other, bigger, frameshifts can also lead to in-frame EGFP. UAS, upstream activator sequences. (B) Time-lapse images of the head region of an embryo derived from crossing *Et(E1b:Gal4-VP16)s1101t* fish to *Tg(UAS:H2A-G_23_-EGFP)hu6243* fish. Stochastic expression of nuclear EGFP (green) in cells was observed, and individual cells and their descendants can be traced over time (denoted by the letters A-F in the images). Images 1–4 represent pictures taken around 16, 21, 25 and 31 hpf and correspond to supplementary material Movie 1. (C) (Upper panel) Schematic representation of reporter plm76 in which Myr-TdTomato is placed downstream of a UAS-cassette, followed by a T2A sequence and H2A-G_23_-EGFP, meaning that EGFP is out-of-frame. Transgenic F1 animals [*Et(E1b:Gal4-VP16)s1101t* fish crossed to *Tg(UAS:Myr-TdTomato-H2A-G_23_-EGFP)hu6242*] exhibit cells that express fluorescent membrane-labeled TdTomato and, in a mosaic pattern caused by *in vivo* stochastic frameshifting, EGFP-fluorescent nuclei (lower panels, an enlarged image of the boxed area is shown in the right-hand image).

To broaden the application of stochastic gene activation in the study of zebrafish biology, we adapted it for combined use with the binary Gal4-UAS system. The available number of Gal4-driver lines for the zebrafish community is large and growing, mainly because of enhancer trap screens (Asakawa et al., 2008; Davison et al., 2007; Distel et al., 2009; Scott et al., 2007). In order to be able to exploit this collection of driver lines and to perform lineage tracing in living animals, we established the UAS-effector line *Tg(UAS:H2A-G_23_-EGFP)hu6243*, which stochastically marks individual cells with nuclear EGFP in tissues where Gal4 is expressed. This line carries the construct plm74, in which the coding sequences for histone 2A (H2A) and EGFP were placed downstream of a UAS-cassette. However, a G23 microsatellite was introduced in between H2A and EGFP, such that EGFP is out-of-frame (a schematic overview of the construct is given in Fig. 2A). We crossed this effector line *Tg(UAS:H2A-G_23_-EGFP)hu6243* with a driver line *Et(E1b:Gal4-VP16)s1101t* (Scott et al., 2007), which widely expresses Gal4-VP16 in many tissues, with central nervous system neurons exhibiting the strongest expression (Schoonheim et al., 2010). In the progeny of that cross, we found stochastic expression of nuclear EGFP, which started at the same time (around the 10-somite stage) and location as Gal4-VP16 would be expressed (Fig. 2B; supplementary material Movie 1). Importantly, to show that this nuclear EGFP can be used as a stable marker to trace cells and their descendants in a living animal, we performed time-lapse imaging (Fig. 2B and supplementary material Movie 1), which demonstrated that nuclear EGFP was stably inherited upon cell division and that individual cells, and their descendants, could be monitored over time.

In order to further facilitate cell fate mapping experiments, we also generated fish containing a construct (plm76) that marked all of the tissue in which Gal4 was expressed. This construct also stochastically marked individual cells with nuclear EGFP. We modified a previously described bicistronic reporter construct (Trichas et al., 2007) that encodes a membrane-bound red fluorescent protein (myr-TdTomato), a viral 2A peptide that allows bicistronic expression (Szymczak et al., 2004) and a nuclear green fluorescent protein (H2A-EGFP). Additionally, here, we placed the reporter downstream of a UAS-cassette and replaced H2A-EGFP with H2A-G_23_-EGFP so that the coding sequence of EGFP was placed out-of-frame (see Fig. 2C for a schematic overview of the construct). The transgenic founder fish *Tg(UAS:Myr-TdTomato-H2A-G_23_-EGFP)hu6242* were crossed to *Et(E1b:Gal4-VP16)s1101t* fish. We found that, in the Gal4-expressing tissues of progeny fish, the majority of cells expressed membrane localized TdTomato, some cells were also found to express nuclear H2A-EGFP (Fig. 2C). Individual cells could be distinguished easily by their red fluorescent membranes, and lineage tracing was facilitated by the random cells that were marked with green nuclear H2A-EGFP. We thus describe two UAS-effector lines – *Tg(UAS:H2A-G_23_-EGFP)hu6243* and *Tg(UAS:Myr-TdTomato-H2A-G_23_-EGFP)hu6242* – that can be used to perform cell fate mapping experiments in Gal4-expressing tissues in living animals.

### Mosaic labeling with self-maintaining Kaloop

**Fig. 3.**
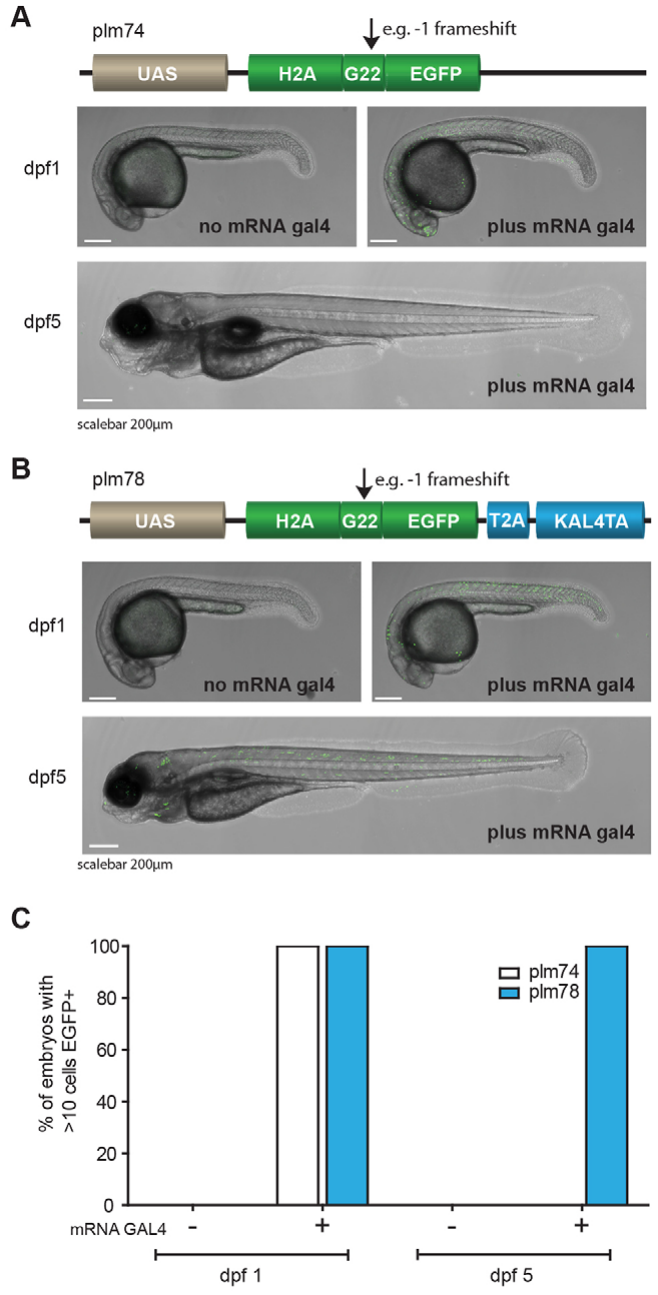
**Mosaic labeling with self-maintaining Kaloop.** (A) Schematic representation of plm74 (upper panel) in which a −1 frameshift at a G_23_ microsatellite results in an ORF of H2A-G22-EGFP. The reporter was injected into one-cell stage embryos with or without *gal4* mRNA to temporarily activate the reporter. Stochastic expression of nuclear EGFP was observed 1 day after injection (right image) when *gal4* mRNA was co-injected, but not in the absence of *gal4* mRNA (left image). EGFP expression diminished within 5 days (lower image; quantified in C). (B) Schematic representation of plm78 in which, owing to a −1 frameshift, the coding sequence of EGFP, together with the linker peptide T2A and transcription activator KalTA4, becomes in-frame with the ORF of H2A-G22. Co-injection of *gal4* mRNA resulted in stochastic activation of nuclear EGFP in embryos at 1 dpf, and expression was still observed in larvae at 5 dpf. (C) Quantification of injected embryos (*n*=100 per condition) that express nuclear EGFP.

In order to follow the fate of all descendants of a single cell, permanent cell-labeling is required to avoid loss of signal when, for example, a promoter is only temporarily activated during embryogenesis. Indeed, a recognized drawback of the binary Gal4-UAS system is that cells and their descendants lose their label once the Gal4-expression is diminished (which depends on the tissue-specific promoter that drives Gal4 expression). This problem has been addressed in a recent report (Distel et al., 2009) through the establishment of a sophisticated self-maintaining system termed ‘Kaloop’. In that study, a bicistronic reporter system was used that included KalTA4 (an optimized version of the Gal4-activator) downstream of a 4×UAS-cassette with EGFP and a T2A sequence, allowing expression of two proteins from the same single transcript (Szymczak et al., 2004). Once activated, KalTA4 maintains its own expression, and that of EGFP, through a positive-feedback loop, because KalTA4 binds in *cis* to its own UAS-promoter and leads to labeling of the complete cell lineage (Distel et al., 2009). We reasoned that lineage tracing by microsatellite instability using Gal4-UAS genetics can be further complemented when combined with the Kaloop system, so that a single cell and its descendants can be followed, even when driver-dependent Gal4 expression is lost. To test this, we adapted plm74 (UAS:H2A-G_23_-EGFP) by placing the coding sequence of KalTA4 and a viral 2A sequence in the same reading frame as that of EGFP (see Fig. 3B for a schematic representation of this reporter; herein termed plm78). After a frameshift mutation, KalTA4 should maintain its own expression, as well as that of H2A-EGFP. Embryos that had been injected with *gal4* mRNA with plm74 or plm78 displayed stochastic labeling with EGFP of individual nuclei at 1 day post-fertilization (dpf) (Fig. 3A,B). Strikingly, embryos that had been injected with plm74 and *gal4* mRNA progressively lost EGFP expression, and at 5 dpf, nuclei that had been labeled previously were undetectable. By contrast, embryos that had been injected with plm78 and mRNA *gal4* maintained EGFP labeling of individual nuclei until, at least, 5 dpf (when animals were killed), indicating that self-maintained labeling of the cells had been established by the Kaloop system. Embryos that had been injected with plm74 without *gal4* mRNA did not show any expression of H2A-EGFP (Fig. 3A, left image). In six out of 100 embryos that had been injected with only plm78 (without co-injection of *gal4* mRNA), we observed a few cells that expressed H2A-EGFP, suggesting self-activation of the Kaloop construct. These results suggest that we have established a UAS-effector line that mosaically labels individual cells by harnessing microsatellite instability. Use of the Kaloop system maintains cell labeling, even when initial driver-dependent Gal4 expression is diminished, and optimizes cell fate mapping for Gal4-driver lines.

### Stochastic activation of Gal4-VP16

**Fig. 4.**
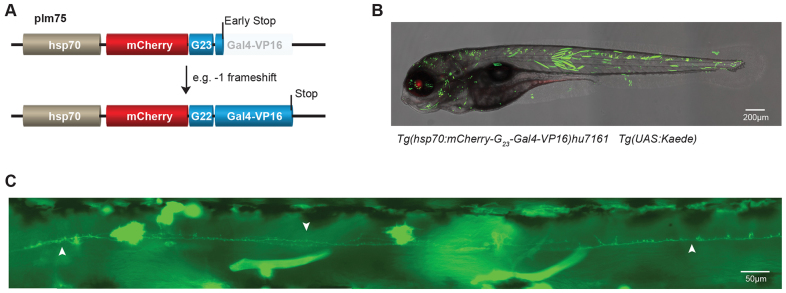
**Stochastic activation of Gal4-VP16.** (A) (Upper panel) Schematic representation of plm75 in which the coding sequence of Gal4-VP16 is placed downstream of that of mCherry, but out-of-frame because of a G_23_ intervening sequence, all under the control of the heat shock *hsp70* promoter. (B) A representative (merged) image shows stochastic expression of Kaede in an embryo at 5 dpf from a cross of the transgenic line *Tg(hsp70:mCherry-G_23_-Gal4-VP16)hu7161* with *Tg(UAS:Kaede)*. (C) Image of a single neuron that was activated stochastically (indicated with white arrows).

In addition to Gal4 driver-lines, many UAS-effector zebrafish lines have become available. To establish a line that can stochastically activate these effector lines through microsatellite instability, we used a similar approach to that described above – the coding sequence for Gal4-VP16 was placed out-of-frame behind mCherry and a microsatellite of 23 guanines (Fig. 4A). The construct was placed downstream of a heat-shock-inducible promoter (*hsp70*) and used to establish the stable transgenic line *Tg(hsp70:mCherry-G_23_-Gal4-VP16)hu7161* that was crossed with a UAS-Kaede line. Without heat shock, mCherry expression was observed in the lens (supplementary material Fig. S2, second left panel); therefore, transgenic animals could easily be recognized and selected. A similar observation has been made previously when using the same promoter (Blechinger et al., 2002; Boniface et al., 2009). After heat shock, we observed strong mCherry expression in the lens and weak, but detectable, expression of mCherry in other embryonic tissues (supplementary material Fig. S2, second right panel). Importantly, in the same embryos, we identified mosaic expression of Kaede (Fig. 4B), often providing clear single cell resolution (Fig. 4C). These data show that the *Tg(hsp70:mCherry-G_23_-Gal4-VP16)hu7161* line can be used to stochastically activate UAS-effector lines through microsatellite instability.

### Sporadic tumor induction in zebrafish

**Fig. 5.**
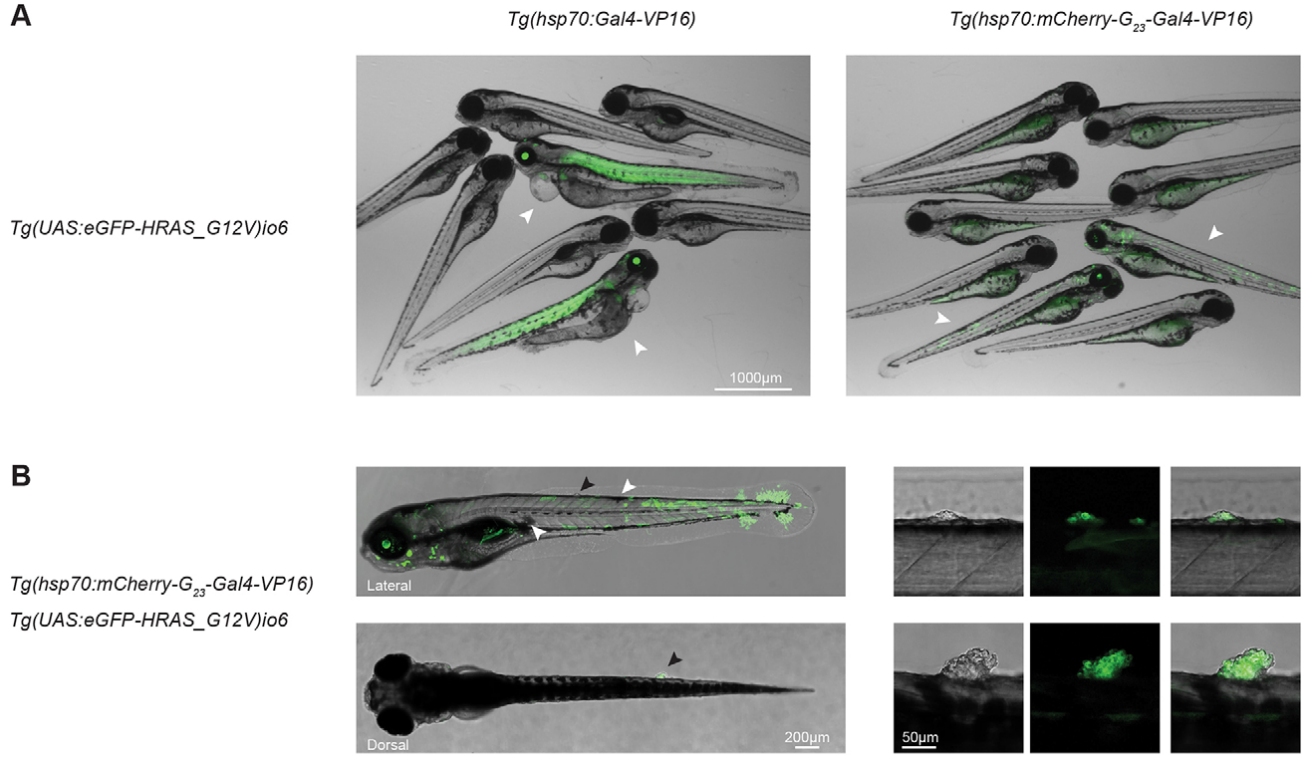
**Sporadic tumor induction.** (A) Merged images show the difference between the expression of UAS:EGFP-HRAS-G12V in a tissue-wide (left) and stochastic (right) manner in embryos; embryos die at 3 dpf upon tissue-wide overexpression of EGFP-HRAS-G12V using *hsp70:Gal4* as an activator, whereas embryos with stochastic activation of EGFP-HRAS-G12V are healthy. White arrowheads indicate embryos that are transgenic for both alleles (the driver and effector alleles), other embryos carried neither of the alleles, or only one of them. (B) Lateral (top images) and dorsal (bottom images) view of a larvae (5 dpf) with stochastic activation of EGFP-HRAS-G12V. Hyperpigmentation (white arrowheads) and tumor formation (black arrowheads) are indicated. Right panels are enlarged images of the areas indicated by the black arrowheads. Brightfield, EGFP and merged channels are displayed.

Modeling human cancers in animals has yielded important findings, and sophisticated murine models have been established to study tumor development. The zebrafish is an emerging model organism in which to study tumorigenesis, mainly because of its ease of *in vivo* imaging and drug screening possibilities. Importantly, pathways that are involved in tumorigenesis – i.e. cell proliferation, angiogenesis, apoptosis – are highly conserved between human and zebrafish, and a wide range of cancers that resemble human malignancies have been identified in zebrafish (Amatruda and Patton, 2008). In many studies, tumor-induction is established through overexpression of an oncogene under the control of a tissue-specific promoter, resulting in tissue-wide overexpression of the oncogene. The current dogma for carcinogenesis is that tumor development starts with a genetic event (often caused by a replication defect) in a single cell that is surrounded with normal cells within that tissue. We reasoned that activation of an oncogene through microsatellite instability *in vivo* should better mimic sporadic carcinogenesis because it is stochastic, cell-division-dependent and restricted to individual cells. To demonstrate the importance of stochastic activation, we compared embryos that overexpressed a constitutively active form of the human oncogene H-RAS in complete tissues or stochastically. For these experiments, we made use of the transgenic line *Tg(UAS:EGFP-HRAS-G12V)io6* (Santoriello et al., 2009), which carries a glycine to valine mutation (G12V) in H-RAS. This mutation locks the protein in an active state and is a common mutation found in patients with the rare genetic disease Costello syndrome (Costello, 1977). H-RAS was tagged with EGFP at the N-terminus in order to visualize cells that express oncogenic H-RAS (Santoriello et al., 2009). First, we used the driver line *Tg(hsp70:Gal4)* (Scheer and Campos-Ortega, 1999) to overexpress EGFP-HRAS-G12V in complete tissues after heat shock. F1 embryos showed, predominantly, strong expression of EGFP-HRAS-G12V in the lens and trunk muscle cells, and most embryos died within 72 hours (Fig. 5A, left panel), which constrained further analysis. To exclude that the heat shock caused the lethality, we also subjected embryos from the same clutch that were not double transgenic to heat shock; all embryos appeared normal, indicating that the lethality that was observed in double transgenic animals was, indeed, owing to the overexpression of EGFP-HRAS-G12V. Next, we crossed the driver line *Tg(hsp70:mCherry-G_23_-Gal4VP16)hu7161* with *Tg(UAS:EGFP-HRAS-G12V)io6* and subjected the F1 embryos to heat shock. Stochastic activation of EGFP-HRAS-G12V was observed in animals that had been heat shocked and, in contrast with larvae from a cross with hsp70:Gal4, the larvae appeared healthy. Interestingly, within 5 days, we observed hyperpigmentation (Fig. 5B, white arrowheads; Table 1; supplementary material Fig. S2) – an early sign of melanoma development (Santoriello et al., 2010). Furthermore, we observed the abnormal growth of cells, which indicated the early onset of tumor formation in 47 out of the 130 embryos that we analyzed (Fig. 5B, black arrowheads and insets; Table 1). All of the early tumors that were observed were EGFP-positive, strongly suggesting that expression of EGFP-HRAS-G12V was driving the abnormal growth of these cells. Additionally, non-heat-shocked animals that were transgenic for EGFP-HRAS-G12V and hsp70:mCherry-G_23_-Gal4-VP16 did not show any tumor formation or hyperpigmentation, again indicating that the overexpression of the oncogene was driving these processes (Table 1). Taken together, these data indicate that, by using microsatellite instability, we activated oncogenic H-RAS-G12V in individual cells, resulting in hyperpigmentation and the onset of sporadic tumor formation. These results highlight the benefit of the stochastic activation of oncogenes in tumor models because the competition between mutant cells and their surrounding wild-type cells can be easily monitored in the same tissue of a single organism, which is impossible when tissue-wide activation of oncogenes is employed.

**Table 1.**
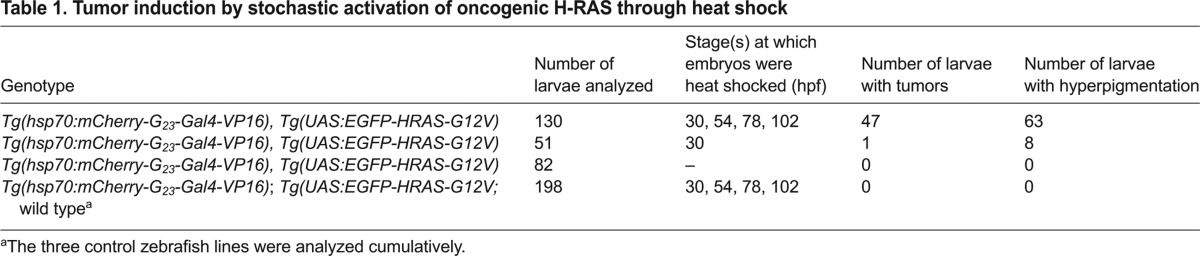
Tumor induction by stochastic activation of oncogenic H-RAS through heat shock

## DISCUSSION

Here, we describe an alternative approach to create mosaicism in zebrafish, which depends on microsatellite instability and avoids labor intensive invasive techniques and limitations that are associated with techniques that require the administration of drugs (Emelyanov and Parinov, 2008; Esengil et al., 2007; Gerety et al., 2013; Hans et al., 2009; Hans et al., 2011; Knopf et al., 2010). Furthermore, we designed our system in a way that it is fully compatible with the large available collection of Gal4-driver and UAS-effector lines.

Although we optimized our approach for the binary Gal4-UAS system, stochastic expression of any gene is possible when the coding sequence is placed behind a microsatellite. Our constructs are designed such that the promoters and coding sequence can be exchanged with relative ease. Additionally, the length of the microsatellite can be altered. Because the frequency of frameshifts depends on the length of the microsatellite (for an example, see Koole et al., 2013), it is possible to increase or decrease the level of stochastic events when varying the tract-length of the microsatellite.

Microsatellite stability is greatly affected by the status of the mismatch repair (MMR) pathway (for a recent review about MMR, see Jiricny, 2013), and, as illustrated in supplementary material Fig. S1, the established lines that contain a microsatellite-reporter offer the potential to study MMR *in vivo*. A study in *C. elegans* using a similar type of reporter has been valuable in the identification of novel genes that are involved in microsatellite instability, allowing the screening of animals that showed enhanced activation of a microsatellite instability reporter (Pothof et al., 2003). Our established transgenic lines, which use microsatellite instability as a read out, provide the potential to find possible new alleles that are involved in MMR when using forward genetic screens, to test candidate genes by reverse genetics approaches – for example, using morpholinos or CRISPR technology (Hwang et al., 2013) – to test MMR-related compounds or to investigate in whole animals those tissues or cells that are more prone to microsatellite instability. These opportunities to study MMR and microsatellite instability *in vivo* in zebrafish is an attractive approach to gain new insights into the MMR-associated Lynch syndrome.

Microsatellite instability is dependent on replication, and thus frameshift mutations can also occur in replicating germ cells. One possible concern is the inheritance of a germline frameshift event that results in progeny having full expression of the transgene in all cells instead of mosaic expression. During the course of our experiments and maintenance of our transgenic lines we found one fish (out of ~250 analyzed) that inherited a germline event and gave rise to embryos in which all cells were labeled. We reason that the frequency of such germline events is low and, therefore, should not provide complications for the maintenance of stable lines over many generations.

In this study, we used microsatellite-dependent activation of oncogenic EGFP-HRAS-G12V to mimic the initial steps of tumorigenesis. Mosaic analysis through microsatellite-dependent oncogene activation has several advantages over other available strategies. First, microsatellite instability is dependent on cell division, and therefore oncogene activation only occurs in proliferative cells. Because ‘driver mutations’ are required for tumorigenesis (Vogelstein et al., 2013) and DNA is most vulnerable to mutations during replication (Aguilera and García-Muse, 2013), it is generally believed that most tumors arise owing to a mutational event in proliferative cells. Other techniques that stochastically activate genes also activate non-dividing cells, which might interfere with accurate reproduction of the early steps of tumorigenesis. Second, microsatellite-dependent activation is restricted to single cells; the chance that a neighboring cell obtains a frameshift mutation at the same time is negligible. This ensures that groups of activated cells are clonally derived. When using other techniques, for example treatment with 4-OHT, there is a chance that neighboring cells are also affected at the same time, which can complicate clonal analysis. Finally, combining this microsatellite instability technique with other techniques allows for the design of new experimental setups. For example, most tumors contain at least two ‘driver’ mutations (Vogelstein et al., 2013), and in order to be able to model two consecutive stochastic events, it is desirable that both events can be induced by two separate techniques that do not interfere with each other.

Modeling sporadic cancers with the help of microsatellite-dependent gene activation has also proven valuable in mouse models (Akyol et al., 2008; Miller et al., 2008). In those studies, the coding sequence of Cre was placed downstream of a microsatellite, enabling stochastic bi-allelic inactivation of floxed tumor suppressor genes or the activation of oncogenes. Although inducible inactivation of tumor suppressor genes is still restricted in zebrafish, we show that stochastic activation of oncogenes is possible in zebrafish. Interestingly, we also show that tumors can be observed easily within days, whereas the timeframe in which tumor development is studied in mice is usually weeks up to months. This temporal advantage, together with the relatively low cost of fish maintenance, the development of high-throughput screening and imaging techniques (Pardo-Martin et al., 2010) to test small molecule libraries (Stern et al., 2005) and the ease of imaging tumor development, makes zebrafish an attractive model in which to study cancer development and perform related drug discovery *in vivo*.

The first mosaic experiments that were employed in zebrafish used invasive techniques; however, in recent years, several genetic non-invasive techniques have been developed to improve the mosaic labeling of cells in zebrafish. Because all techniques have advantages and limitations, it is of great importance that scientists have a variety of tools from which they can choose (and then combine) to best suit their experiments. The use of the microsatellite-dependent activation of transgenes will expand the ‘toolbox’ for mosaic analysis in zebrafish and provide new opportunities to perform cell lineage tracing experiments, gene function studies and research into tumor biology.

## MATERIALS AND METHODS

### Plasmid construction

All plasmids comprised six elements (with the exception of plm78 and plm76) – (1) the pminiTol2 plasmid (pDB739) as backbone (Balciunas et al., 2006); (2) a *hsp70* promoter (Halloran et al., 2000) or 14×UAS sequences, both flanked with a *Swa*I and a *Kpn*I restriction site; (3) a coding sequence (e.g. mCherry, EGFP or H2A) starting with a Kozak sequence but lacking a stop codon and flanked with a *Kpn*I and an *Nhe*I restriction site; (4) a microsatellite or random sequence (5′-GATTCTGCCAAGT-3′) with flanking *Nhe*I and *Bam*HI restriction sites; (5) a coding sequence [EGFP or Gal4-VP16 (Köster and Fraser, 2001)] without a start codon but including a stop codon that was flanked with *Bam*HI and *Xba*I restriction sites; (6) a SV40 3′UTR flanked with *Xba*I and *Hin*dIII restriction sites. Elements two, three and five were obtained through PCR-amplification, element four was obtained through the cloning of DNA oligos. All elements allow easy substitution because most of the flanking restriction sites are unique. Plm78 was created by swapping KGFP from the plasmid 4×Kaloop (pTolmini-4xUASKGFP-T2A-KalTA4GI) (Distel et al., 2009) with H2A-G_23_-EGFP using the restriction sites *Brg*I and *Eco*RI. To create plm76, we used the construct Tom-2A-GFP (Trichas et al., 2007) in which we replaced the promoter with a UAS cassette, and H2B-GFP with H2A-G_23_-EGFP.

### Fish maintenance, transgenesis and transgene-induction

Wild-type and transgenic embryos were obtained by natural spawning of adult fish that were maintained at 28.5°C. For transgenesis, one-cell stage embryos (F0) were co-injected with 5–20 pg of plasmid DNA and 20 pg of transposase mRNA, as described previously (Balciunas et al., 2006; Kawakami et al., 2004; Kawakami, 2005). Injected embryos were grown until adulthood, crossed with Gal4- or UAS-lines, and the F1 progeny were examined by fluorescent microscopy. Positive embryos were selected and raised. For heat-shock treatment, embryos were transferred to a 50 ml tube and heat shocked for 30 minutes at 37°C in a waterbath.

### Microscopy

For microscopy, embryos were anesthetized with Tricaine (Sigma) and fixed using 0.5% low-melting-point agarose in glass-bottomed dishes (MatTek). Images were taken by using a Leica TCS SP5-STED microscope. Image stacks were taken with a 10× objective and 1.3 digital zoom. For time-lapse imaging, embryos (kept in the chorion) were fixed in 0.5% low-melting-point agarose, and a heated-stage chamber was used to keep embryos at 28.5°C. Image stacks were taken every 25 minutes. Images were processed using Leica software for automatic stitching and ImageJ to create overlays and maximum projections.

### X-gal staining

Embryos were heat shocked for 30 minutes at 37°C, left to recover for 6 hours, fixed on ice for 30 minutes in freshly prepared fixing solution (PBS, 1% formaldehyde, 0.2% gluteraldehyde, 0.02% IGEPAL) and then washed three times for 20 minutes in PBS at room temperature. Next, the embryos were stained for β-galactosidase expression overnight in X-gal solution (0.16 M Na_2_HPO_4_, 0.03 M NaH_2_PO_4_, 0.2 mM MgCl_2_, 0.1 mM SDS, 5 mM [Fe(CN)_6_]^3−^, 5 mM [Fe(CN)_6_]^4−^, 1 mM X-gal). All fixation, washing and staining steps were performed on a shaking platform.

### Tumor induction

To induce expression of EGFP-HRAS-G12V, we heat shocked embryos (which had been transferred to a 50 ml tube) for 30 minutes at 37°C at 30 hpf, 54 hpf, 78 hpf and 102 hpf, unless stated otherwise.

## Supplementary Material

Supplementary Material
