# Supplementary material for: Mosaic analysis and tumor induction in zebrafish by microsatellite instability-mediated stochastic gene expression
Source: Dis Model Mech. 2014 Jan 30;7(7):929–36. doi: 10.1242/dmm.014365 (PMC4073281; doi:10.1242/dmm.014365)
Supplement: Supplementary Material [file supp_7_7_929__index.html]

Mosaic analysis and tumor induction in zebrafish by microsatellite instability-mediated stochastic gene expression — Supplementary Material 

# Mosaic analysis and tumor induction in zebrafish by microsatellite instability-mediated stochastic gene expression

## DMM014365 Supplementary Material

**Files in this Data Supplement:**

- **Supplementary Material**
