# Supplementary material for: Mosaic analysis and tumor induction in zebrafish by microsatellite instability-mediated stochastic gene expression
Source: Dis Model Mech. 2014 Jan 30;7(7):929–36. doi: 10.1242/dmm.014365 (PMC4073281; doi:10.1242/dmm.014365)
Supplement: Supplementary Material [file supp_7.7.929_DMM014365.pdf]

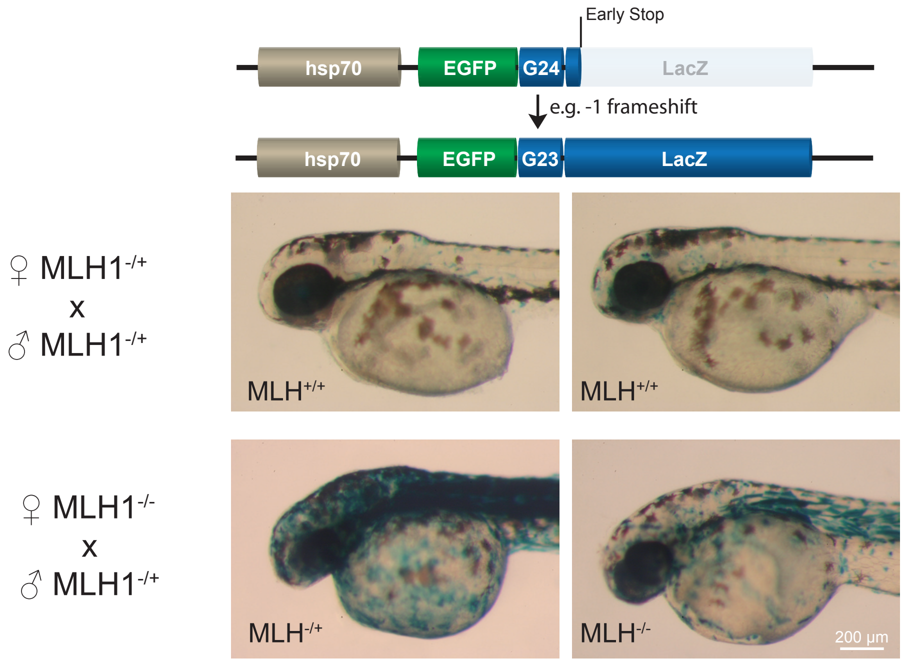

Supplementary figure 1 Increased microsatellite instability reporter activation in mismatch repair deficient embryos.

Images of embryos (2dpf) stained for LacZ expression. One-cell stage embryos were made transgenic via Tol2-transposase mediated transgenesis with the indicated microsatellite instability construct that reports LacZ expression when the LacZ-coding sequence becomes in-frame after a frameshift. Strong LacZ-staining (examples shown in bottom panels) was seen in 20 out of 22 injected viable embryos that were obtained from a cross between an $MLH1^{-/-}$ mother and an $MLH1^{-/+}$ father. Genotyping of animals showed that also heterozygous embryos from this cross had strong LacZ-staining, suggesting that the compromised mismatch repair pathway (due to MLH1-deficiency in the yolk) is not rescued by the paternal MLH1 wildtype allele during early development. Injected embryos obtained from $MLH1^{-/+}$ parents, showed LacZ-staining similar to wild type levels (top panels and Fig. 1).

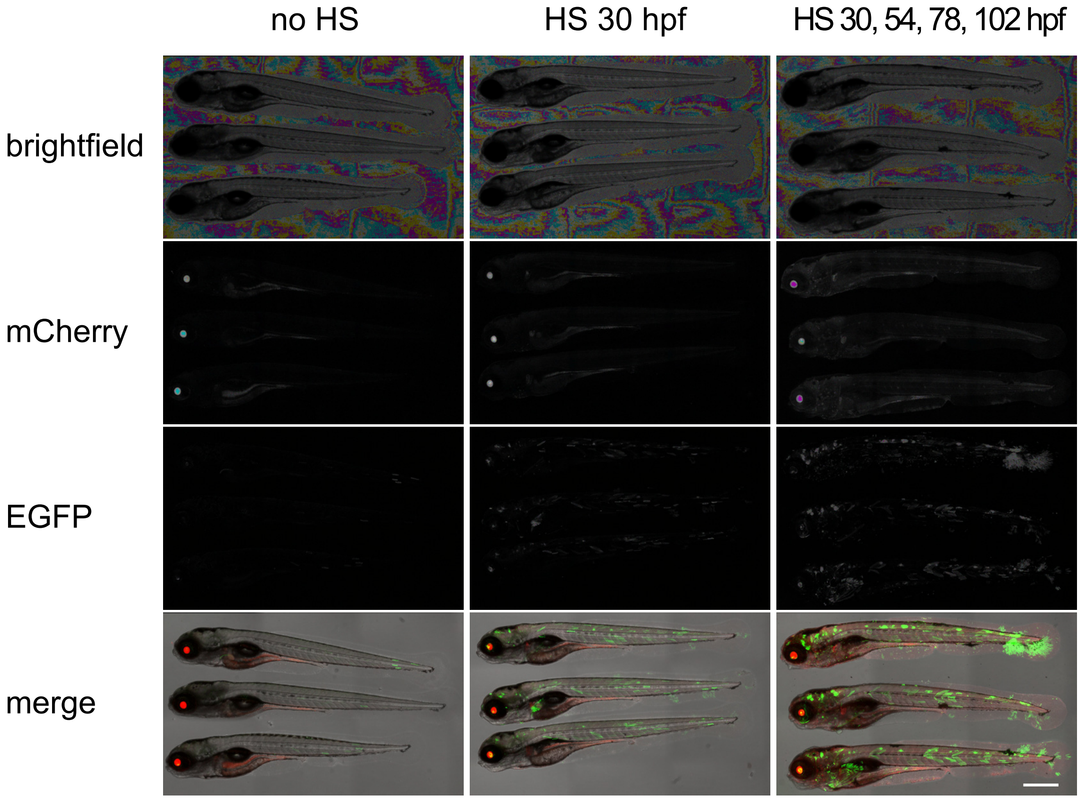

Supplementary figure 2 Random activation of oncogenic H-RAS by stochastic Gal4 expression.

Larvae were heat shocked (HS) at various time points, as indicated. Larvae were derived from a cross *Tg(hsp70:mCherry-G*$_{23}$*-Gal4VP16)hu7161* with *Tg(UAS:EGFP-H-RAS_G12V)io6*. Larvae subjected to heat shock treatment showed stochastic expression of EGFP-H-RAS(G12V), hyper-pigmentation and tumor formation. The level of these phenotypes was depended on the number of heat shock treatments (see Table 1 for quantification). Images were taken at 126 hpf. Scalebar indicates 0.5 mm.

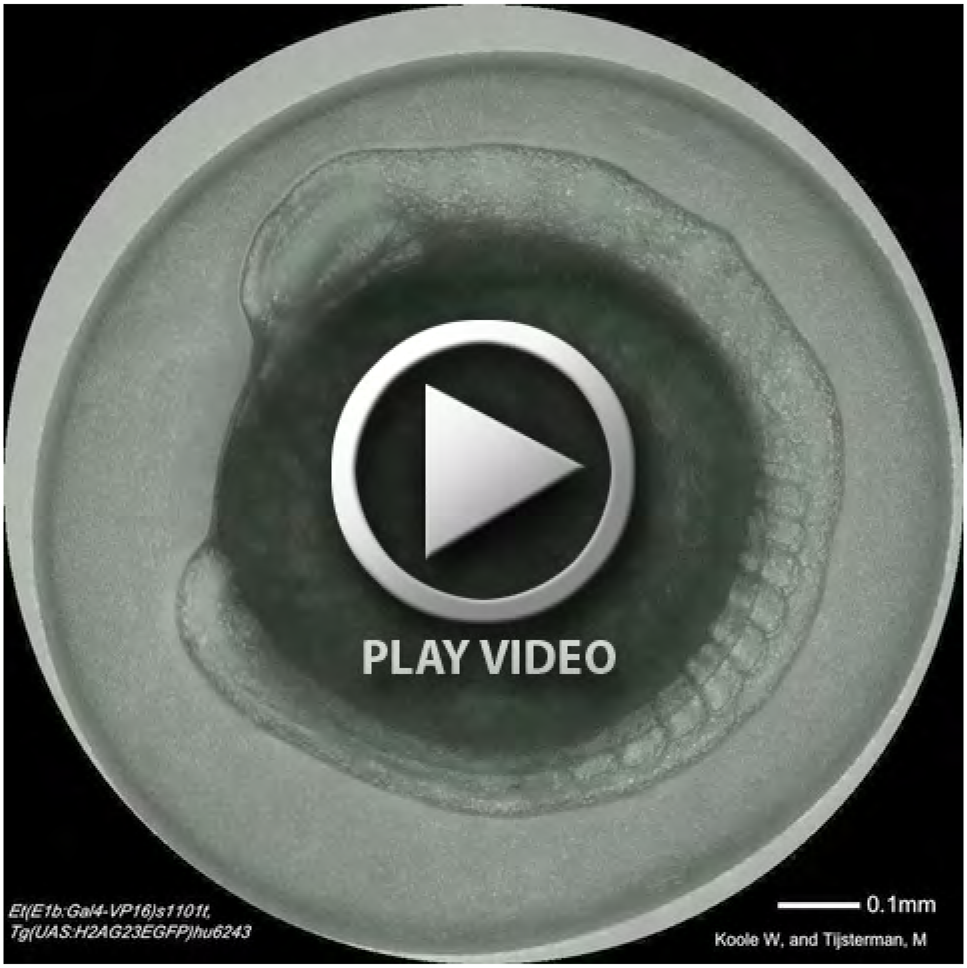

Supplementary Movie 1. Mosaic labeling of cells with nuclear EGFP through microsatellite instability. Time-lapse movie demonstrating the “birth” of EGFP marked cells, in which H2A-EGFP was stochastically activated through microsatellite instability. After being recognized, the mobility of these cells as well as their descendants can be followed in time in live animals. Time-lapse images were taken from approximately 14hpf untill 45hpf. A H2A-EGFP positive cell was marked with an arrow to illustrate that after division daughter cells can be followed over time. The genotype and scalebar are indicated in left and right bottom corner of the movie.
